# Beyond first impressions: the importance of seeing the whole person

**DOI:** 10.1038/s41390-024-03372-4

**Published:** 2024-07-11

**Authors:** Meg Raby, Michele Kong

**Affiliations:** 1 KultureCity, Birmingham, AL USA; 2 The University of Alabama at Birmingham, Birmingham, AL USA

## Abstract

The power of a first impression and the trajectory it can set an individual on is something that impacts all people, and especially those with invisible disabilities. Societal norms and pragmatics are centered around neurotypical individuals and do not consider the perspective and neurology of the neurodivergent. What is the social norm for society can literally be impossible or physically uncomfortable for the neurodivergent, resulting in a mismatched doctor-patient relationship, an unintentionally inaccurate plan of care, or even a missed chance at a successful hire. We are presently in the neurodivergent movement where intentional inclusive practices are happening. First impressions coupled with curiosity and a better understanding of neurodivergence, could be the answer to a more positive relationship and outcome for all, no matter the situation or environment.

First impressions are everything. One’s appearance, energy, scent, communication style and the way they greet or don’t greet can be a make-or-break interaction that dictates the rest of that person’s life. People tend to let others who make them feel respected and comfortable into their social circles. They want to be around people that make them feel good, and they don’t want to be around people that make them feel uneasy. But what if the reason for the not-so-comfortable exchange was due to a neurology that translates information in a different way? What if the person being interacted with has a brain that processes external and internal stimuli in a way that makes it difficult to follow common rules of social engagement? What if they are a non-speaker, not using verbal speech to express themselves and to interact?

One in four individuals in the United States have a disability, and often times invisible, including those with Attention-deficit/hyperactivity disorder (ADHD), autism, posttraumatic stress disorder (PTSD), anxiety and panic disorders, Parkinson’s, dementia and recovery from stroke to name a few.^1^ Something as common as grocery shopping or riding in an elevator can turn into an overstimulating endeavor that leaves these individuals feeling dysregulated. When the senses, either one or multiple at a time, become flooded with information or are lacking in input, focusing in and accessing needed information to process and navigate what is happening, or utilizing speech and language in a cohesive manner can be nearly impossible. These can lead to erratic behaviors and a disconnected disposition, which often results in disapproving looks from surrounding bystanders, compounding the already overwhelming situation.

Those who have a sensory processing impairment or difference are also at an increased likelihood of being a non-speaker. A non-speaker is an individual who primarily uses alternative forms of communication rather than the speech mechanism. It is estimated that over 97 million humans worldwide are non-speaking and could benefit from an alternative means of communication that integrates technology.^2^ For non-speakers, it is often assumed that because they are not able or do not use verbal speech, that this correlates to a lower cognitive ability. Such an assumption leads to a first impression that will surely impact the interaction via inherent biases. It is important to understand that a stance of presuming cognitive competence is integral when communicating with non-speakers, and for the appropriate methods of communication to be used. It was recently reported that up to five times more non-speaking autistic teens and adults have a knowledge bank of written language conventions than what is expected of their abilities.^3^

In the healthcare setting, first impressions can sometimes become the very stumbling block in patient diagnosis or management if it inadvertently becomes the lens through which the assessment is done. Take for instance, *Emily who presented to the emergency room with head hitting. Her chart revealed that she was autistic, and non-speaking. The physician made a diagnosis of viral illness and discharged her home based on the constellation of fever, malaise, and otherwise unremarkable lab work. His first impression of her being an autist who was ‘stimming’ proved to be grave, as Emily suffered a cardiac arrest at home due to encephalitis. Had he asked about Emily as a person, her baseline behaviors, her likes and dislikes and methods of communication, he would have uncovered that the head hitting was a new symptom, and not her ‘stim’, and that although a non-speaker, she communicated with a device, which she was not able to do because her mental status was compromised. First impressions can have a substantial impact on outcome; medical armamentarium can guide the healthcare provider on what to treat and how to treat, but without a comprehensive understanding of the neurodivergent patient, many who have sensory processing impairment, or those who are non-speaking, the interactions and trajectory of care is going to be much more piecemealed rather than thorough and cohesive.

First impressions also have the potential to lead to loss of patient autonomy, and an overall negative experience, as evidenced by this office encounter. *Greta, a teenager who was non-speaking entered the exam room for the first time with her mother, an iPad in hand. Bright sunlight casted into the room, causing Greta discomfort from the visual input and a need to ‘stim’ to soothe and regulate. She began swaying back and forth, and moving her head from side to side, squinting. The doctor entered the exam room, looked at Greta, but proceeded to shake the mother’s hand first, introducing himself and then said hello to Greta. Before Greta was able to form her response using her iPad and before she was back to homeostasis–where her senses were not overwhelmed, he asked the mother, “What brings her here today?” and with that Greta’s competency and autonomy over her healthcare was immediately negated. The doctor had innately decided that she was not able to provide the information he needed to treat her and made the decision to speak with Greta’s mother instead.

Or take *Marie’s recent experience at a medical checkup. Marie was an accomplished writer, married and a mother. She greeted the doctor warmly, and after a few minutes of small talk, he discovered that she was autistic. He blurted out, “You’re autistic? Well, not that autistic…and you certainly don’t want or need to tell anyone. That could impact your employment opportunities.” Because the doctor was quick to make assumptions about Marie and about what it meant to be autistic, Marie felt uncomfortable and sought further medical help elsewhere.

First impressions matter in all aspects of our lives. Even in the hiring domain, it is perhaps one of the key factors in determining an individual’s chances at making the cut for a position. The natural anxiety born of an upcoming job interview coupled with a neurodivergent individual’s social, communication and sensory barriers can led to a scenario where one is not truly judged for their abilities or competency.

^*^Joe, a young autist was a classic example of a successful hire because they saw past his socially different behavior. Before his interview began, the hiring director noticed that Joe’s hair was unkempt, and he had a certain scent. During the interview, Joe appeared cold and ignored the director’s extended hand for a handshake. Rather, he jumped right into big data and statistical strategies for the division. The interviewer cracked a joke that didn’t elicit a laugh from Joe and the whole energy felt uncomfortable and “off”. Would that have been the end of Joe’s running as a candidate for the position? For many companies hiring, very likely. In this instance, because of the hiring director’s familiarity with neurological differences, it did not prevent him from seeing Joe for who he truly was. After a second interview, Joe was hired, and he remains one of the top employees in this division today.

In our post-pandemic era of high zoom usage, first impressions made on the screen, presents additional unique challenges for individuals with disabilities. For neurodivergent users, orienting to the speaker on the screen, especially when there are multiple speakers can be a struggle, particularly for individuals with visual impairments or have processing difficulties. Additionally, the absence of closed captioning can be difficult for those who are hard of hearing or have noise sensitivity, making it challenging to follow the conversation accurately. These barriers lead to increased stress and difficulty in effective communication, potentially impacting the outcome of the zoom session, and overall experience.

We are presently in the neurodivergent movement. More is being understood about those who are neurodivergent, and more intention is being placed on gaining further understanding to best accommodate these individuals. But there is much work to be done. The misunderstandings are still present and society at large remains unfamiliar with the unique way that a neurodivergent individual processes our world. We need to see them for who they are and what they’re capable of-for their strengths that undoubtedly exist and their integral contribution to our community that remain untapped due to inaccessibility and accommodations. First impressions really are everything, but perhaps they should be coupled closely with curiosity. Perhaps pausing and considering other factors at play is key to a successful interaction, whether at the hospital, in our workplace, home or community.

^*^Names are modified to maintain anonymity-all stories based on a real-life encounters.
